# Lifestyle Changes among Mexican People during the COVID-19 Lockdown in 2020: A Cross-Sectional Study

**DOI:** 10.3390/healthcare10122537

**Published:** 2022-12-14

**Authors:** Rocio Guadalupe Hernández-Nava, María de la Luz Sánchez-Mundo, Raquel García-Barrientos, Vicente Espinosa-Solis, Patricia Villalobos-Aguayo, Nancy Natividad Salmerón-Muñiz, José Daniel Anaya-Tacuba

**Affiliations:** 1 Escuela Superior de Nutrición y Ciencia de los Alimentos Campus Llano Largo, Universidad Autónoma de Guerrero, Carretera Cayaco-Puerto Márquez Parcela 56, 57 y 58, Acapulco 39906, Guerrero, Mexico; 2 Instituto Tecnológico Superior de Las Choapas, Tecnológico Nacional de México, Carretera Las Choapas-Cerro de Nanchital Km 6.0 Col. J. Mario Rosado, Las Choapas 96980, Veracruz, Mexico; 3 Laboratorio de Procesos Biotecnológicos, Universidad Politécnica de Tlaxcala, Avenida Universidad Politécnica No. 1, Tepeyanco 0180, Tlaxcala, Mexico; 4 Coordinación Académica Región Huasteca Sur, Universidad Autónoma de San Luis Potosí, Km 5, Carretera Tamazunchale-San Martin, Tamazunchale 79960, San Luis Potosí, Mexico; 5 Facultad de Medicina, Universidad Autónoma de Guerrero, Av. Solidaridad s/n, Acapulco 39350, Guerrero, Mexico; 6 Escuela Superior de Ciencias Naturales, Universidad Autónoma de Guerrero, Carretera Nacional Chilpancingo, Petaquillas, Ex Rancho Shalako, Chilpancingo 39105, Guerrero, Mexico

**Keywords:** eating habits, COVID-19 lockdown, Mexican population

## Abstract

The COVID-19 pandemic generated a new challenge of our time with different scenarios. For this reason, this work aimed to identify changes in the diet and lifestyle of Mexicans during the COVID-19 lockdown. This study was based on a Google online survey, which contained questions about eating habits, physical activity, and sleep behavior before and during the COVID-19 lockdown. It was conducted from 2 June to 4 July 2020 and included 1004 participants (698 women and 306 men 18 years old and above). The subjects reported the increased frequency of consumption of meat, vegetables, fruits, eggs, legumes, fast food, and alcoholic drinks during the lockdown. Furthermore, subjects reported sleeping more hours than usual and negative changes in sleep quality (insomnia, nightmares, and leg pain or cramps). There was a reduction in practicing 30 min of intense physical activity during the week. On the other hand, there was an increase in the number of hours spent in front of the screen. Participants over 36 years of age performed less physical activity during the COVID-19 lockdown. These results indicated that it is vital to develop national strategies to promote healthy lifestyle habits in the population during pandemic lockdown measures.

## 1. Introduction

On 11 March, the World Health Organization (WHO, Geneva, Switzerland) declared a COVID-19 pandemic [1]. Lockdown was the first health action taken by several countries to reduce the collapse of health services due to the rapid spread of the SARS-CoV2 virus. The first case of COVID-19 in Mexico was reported on 25 February, and the first death was on 18 March. On 30 March, the Mexican government declared a national health emergency and planned a strategy to lessen its rapid spread. This strategy was the National Campaign of Social Distancing, in which non-essential activities were limited in the public and private sectors, avoiding attendance at work centers and public-crowded places. In addition, some sectors of the Mexican population had to work from home, avoiding going out into the streets. Other actions employed by the Mexican authorities were the closure of schools at all academic levels. Additionally, the learn-at-home program was implemented at the elementary and secondary education levels, which consisted of educational videos broadcast on television as a tool of school reinforcement in the public sector [2,3]. On the other hand, public spaces for physical activity were closed; some people were forced to reduce their physical activity, which can negatively affect sleep quality [4]. Furthermore, physical inactivity, poor sleep quality, and spending more time in front of screens are unhealthy behaviors associated with an increased risk of obesity, which generated more severe conditions due to COVID-19 [5,6].

A healthy balanced diet is essential to establishing a personal risk management strategy during pandemics, such as COVID-19. Several investigations regarding the effect on eating behavior and lifestyle in different countries before and during the COVID-19 lockdown have been conducted to establish how nutrition and lifestyle modifications could help as adjuvant therapy to reduce the risk through enhanced immunity. In China [7] during January and February 2020, the effect of lockdown was investigated, and it was concluded that more than half of the adult Chinese population temporarily adopted a sedentary lifestyle with insufficient physical activity, more screen time, and poor emotional health, which could create a considerable health risk. In the United Arab Emirates [8], it was observed that although the lockdown due to COVID-19 was necessary to maintain public health, it caused changes in lifestyle, physical inactivity, and psychological problems in adults. In Kuwait [9], during the COVID-19 lockdown from 19 June–15 July 2020, different lifestyle changes were reported in men and women. An increase in fruit and vegetable consumption and a decrease in sweetened beverage consumption were reported. Additionally, women increased their total food consumption, and men increased their smoking frequency. In Mexico [10], from 26 April–29 May 2020, an online survey was carried out to investigate food choices and consumption changes in Mexican households during the COVID-19 lockdown. The study showed different attitudes towards food, a trend to reduce the consumption of alcohol and soft drinks, and to increase consumption of vegetables and fruits, while other food categories remained constant. Furthermore, this investigation showed that in middle- and high-income households, the attitudes towards food were related to health, hedonism, and economic aspects. However, these were not related to health status, being overweight, or family size, but were related to the income level of households and, above all, to education. 

The COVID-19 pandemic, combined with global warming, natural resource depletion, and the Russian–Ukrainian war, represents an important challenge we must face today [11]. Therefore, it is essential to know how these new concerns affect the well-being of the world population. So far, there are various reports from different countries on the effect of the COVID-19 lockdown on the population’s lifestyle. No studies about changes in physical activity and sleep behavior of the Mexican population before and during the COVID-19 lockdown have been reported. Therefore, this study aimed to investigate the effect of the COVID-19 lockdown on Mexicans’ eating habits, physical activity, and sleep behavior as affected by social distancing and self-isolation through an online survey.

## 2. Materials and Methods

### 2.1. Survey

This study was designed to collect cross-sectional data using an online survey. An invitation to participate in the survey was sent to the researchers’ contacts through social networks to reach a large convenience sample. The social networks used were FacebookTM (Cambridge, MA, USA), WhatsAppTM (Menlo Park, CA, USA), and Twitter (San Francisco, CA, USA). In addition, the survey was emailed to family, friends, and colleagues with a request to forward the invitation to people interested in participating. Data were collected through the Google forms platform from 2 June to 4 July 2020. A total of 1007 people answered the survey, and three questionnaires were eliminated since persons under 18 years old answered them. So, a total of one thousand and four questionnaires were responded. The minimum number of surveys needed in this study was calculated by considering that the total population in México is 124,014,024 persons, as reported in the 2020 population and housing census performed by the Mexican Institute of Geography and Statistics [12], and using a confidence level of 95% and a margin of error of 5%. According to the calculated data, the sample size is 385. Therefore, in our study, we used almost three times the minimum representative size of the sample (1004) for the analysis of the Mexican population.

### 2.2. Questionnaire

**Figure 1:**
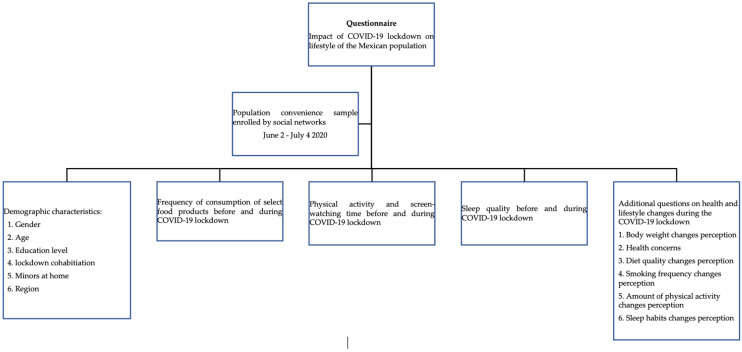
Schematic representation of the study participants and sections of the online questionnaire.

The questionnaire used was designed by a multidisciplinary group of experts, including doctors, nutritionists, public health specialists, and statisticians. Cronbach’s alpha coefficient was used to assess internal consistency reliability. A value of 0.81 was found, ensuring the validity of the questionnaire. On a single occasion, participants answered the survey, which contained questions about eating habits, physical activity, and sleep behavior before and during the COVID-19 lockdown. The questionnaire had four sections (Figure 1). The first section was the food habits questionnaire, where through 15 items, participants were questioned about the frequency of food and beverage consumption before the pandemic and during the lockdown. The answer options were (1) less than once a week (2) consume them 1–2 days a week; (3) consume them 3–4 days a week; (4) consume them 5–6 days a week, and (5) consume them seven days in a week. The second section asked about the frequency of doing 30 min of intense physical activity in a week and the participants’ physical activity before the pandemic and during the lockdown. Additionally, screen time was counted as the number of hours sitting in front of the screen, including work and non-work activities. The third section addressed the sleep assessment; participants were asked to compare their sleep routine before the pandemic and during the lockdown, considering the number of hours and the quality of sleep, asking if they experienced certain types of discomforts such as insomnia, cramps, and grinding of teeth. Additional questions, such as those related to the perception of body weight changes, health concerns, diet quality changes, smoking frequency changes, amount of physical activity changes, and sleeping habit changes, were applied in order to examine the perceived health and lifestyle changes during the COVID-19 lockdown. 

### 2.3. Sociodemographic Characteristics of Participants

The authors designed the questionnaire in which sociodemographic information was considered (age, sex, residence place, educational level, coexistence with other people during lockdown, and coexistence with children during lockdown). The minimum age required to participate in this study was 18 years old. The states belonging to the Mexican Republic were grouped into 8 regions: the northwest region (Baja California, Baja California Sur, Chihuahua, Durango, Sinaloa, and Sonora), northeast region (Coahuila, Nuevo León, and Tamaulipas), west region (Nayarit, Jalisco, Colima, and Michoacán), east region (Puebla, Veracruz, Tlaxcala and Hidalgo), north central region (Aguascalientes, Guanajuato, San Luis Potosí, Zacatecas, and Querétaro), south central region (Morelos, State of Mexico, and Mexico City), southwest region (Guerrero, Oaxaca, and Chiapas), and southeast region (Tabasco, Campeche, Quintana Roo, and Yucatán).

### 2.4. Ethical Concerns

The present study adhered to the Declaration of Helsinki (2008) and Nuremberg (1947), granting respect for individuals, seeking their well-being, the integrity of the human being, and the right to decide to take part or not in the study, without pressure and after having known the pros, cons, and benefits of their participation.

This study contained informed consent about the uses of the collected data, it was obtained anonymously with no indication of any personal information, and participants were not rewarded. The study protocol was approved by the Bioethics Committee at the Autonomous University of Guerrero (CB-008/2K20), Mexico.

### 2.5. Statistical Analyses

Differences in food frequency consumption before and during lockdown were tested using the nonparametric paired-sample Wilcoxon test. The categorical variables are presented as numerical totals and percentages. In addition, The McNemar test was used to determine if differences existed on a dichotomous dependent variable between two related groups to check the differences between the tested variables “before” and “during” the COVID-19 lockdown, while the Chi-Square test was used to examine the differences between categorical variables in the same population to determine associations between the categorical variables with a *p* < 0.05. Statistical analysis was performed with the SPSS version 28.0 program (IBM, Chicago, IL, USA).

## 3. Results

### 3.1. Participants’ Characteristics

In this study, 1004 surveys were answered by 698 women (69.5%) and 306 men (30.5%), 50.8% of participants were from the age group of 18 to 25 years, and 65.5% of the total participants had undergone undergraduate studies. In addition, 77.6% of participants spent the COVID-19 lockdown with their families, where 56.6% of those homes had children, and 60.7% of the participants lived in the southwestern region of Mexico (Table 1).

### 3.2. Frequency of Dietary Intakes of Certain Food Groups

**Figure 2:**
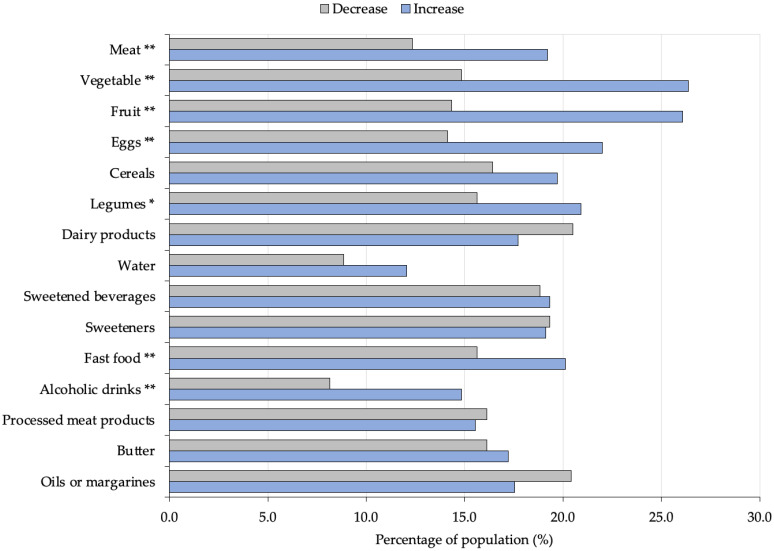
Percentage of population that reported a variation (increase or decrease) in the intake of each foodstuff during the COVID-19 lockdown. People who reported no change in the frequency of consumption are not represented in this figure. Statistically significant differences between food intake before and during the COVID-19 lockdown period were assessed by a Wilcoxon test and are indicated with asterisks (* *p* < 0.05; ** *p* < 0.001).

The consumption frequency of different foods before and during the COVID-19 lockdown of the Mexican population is displayed in the Supplementary Materials (Table S1). As shown in Figure 2, a positive and significant increase in the frequency of consumption of fruit, vegetables (*p* < 0.001), and legumes (*p* = 0.007) was observed. Regarding animal-origin foods, an increase in intake frequency was observed for meat and eggs (*p* < 0.001). The percentage of the population that reported an increase in consumption ranged between 19% and 26% for all these items, all of which were associated with a healthy dietary pattern [13]. On the other hand, no statistically significant changes were found in the frequency of consumption regarding cereals, dairy products, water, sweetened beverages, sweeteners, processed meat products, or butter, oils and margarines. Furthermore, as a negative aspect, the participants surveyed during lockdown significantly increased (*p* < 0.001) their intake of fast food and alcoholic drinks.

### 3.3. Physical Activity and Screen-Watching Time

**Figure 3:**
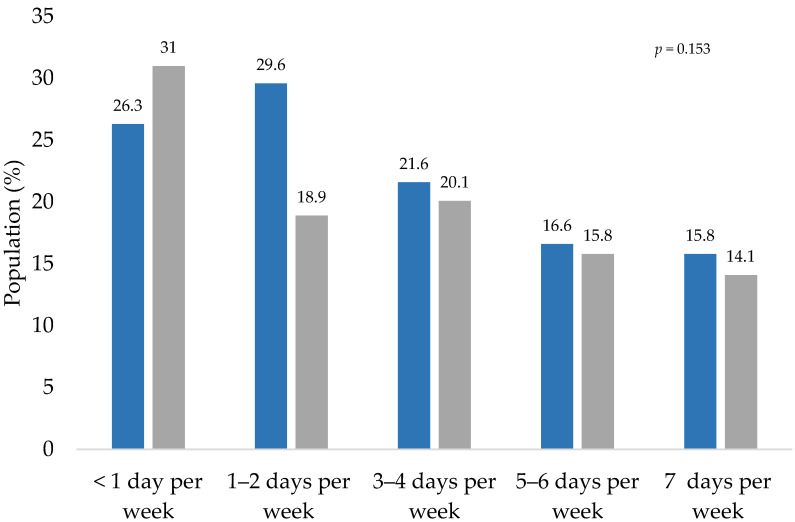
Frequency of 30 min of intense physical activity, before and during the COVID-19 lockdown. Blue bars represent the activity of participants before the lockdown and gray bars represent the activity during the lockdown. The *p*-values indicate the statistical significance of McNemar test.

The frequency with which people performed thirty minutes of intense physical activity is shown in Figure 3. It is observed that Mexicans decreased the level of physical activity during the COVID-19 lockdown.

**Figure 4:**
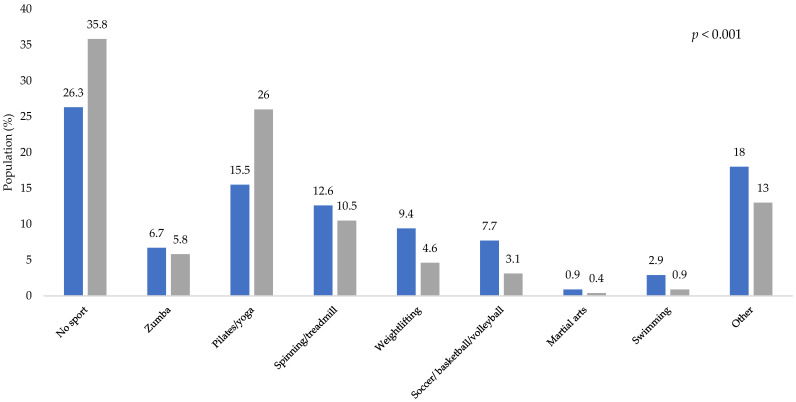
Type of training before and during COVID-19 lockdown. Blue bars represent the activity of participants before the lockdown and gray bars represent the activity during the lockdown. The *p*-values indicate the statistical significance of the McNemar test.

The type of physical activity performed before and during the COVID-19 lockdown is shown in Figure 4. A total of 26.3% of participants reported not doing any physical activity before the COVID-19 pandemic, and during the lockdown, this percentage increased to 35.8% (*p* < 0.001). People who practiced Zumba, bicycle/treadmill, weightlifting, soccer/basketball/volleyball, martial arts, swimming, and other physical activity not mentioned in this study significantly decreased the performance of these physical activities during the lockdown. However, the practice of Pilates/yoga increased by 10.5% during lockdown (*p* < 0.001).

**Figure 5:**
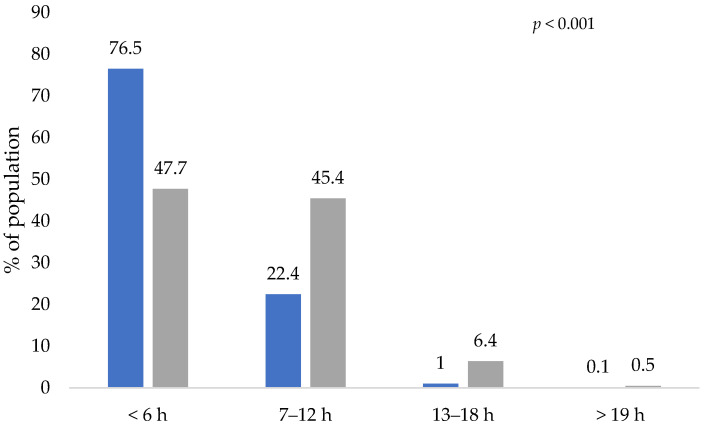
Screen-watching time before and during the COVID-19 lockdown. Blue bars represent the activity of participants before the lockdown and gray bars represent the activity during the lockdown. The *p*-values indicate the statistical significance of the McNemar test.

During the lockdown, respondents spent more hours a day sitting in front of the screen, performing work-related and non-work-related activities (computer, tablet, TV, video, games, reading, social networks, series, etc.). Figure 5 represents the time spent watching the screen (in hours) before and during the COVID-19 lockdown. There was an increase of 1.0% and 6.4% for those who watched the screen 7–12 h/day and 13–18 h/day, respectively.

### 3.4. Sleep Duration and Quality

**Figure 6:**
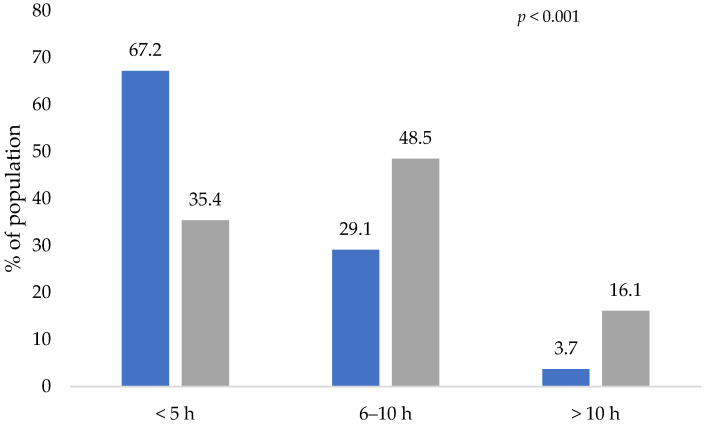
Hours of sleep per night before and during the COVID-19 lockdown. Blue bars represent the activity of participants before the lockdown and gray bars represent the activity during the lockdown. The *p*-values indicate the statistical significance of the McNemar test.

Figure 6 presents the hours of sleep per night before and during the COVID-19 lockdown. During this pandemic, people in Mexico increased the number of sleeping hours per day. During the COVID-19 lockdown, the number of participants who slept from 6–10 h per day and more than 10 h per day increased from 29.1% to 48.5%, and from 3.7% to 16.1%, respectively. Although the participants in this study were sleeping a greater number of hours, they presented changes in sleep quality (Table 2) due to an increase in the percentage of people who suffered from insomnia (28.9%) and nightmares (8.1%), and in those who presented leg pain or cramps during the lockdown (5%) (*p* < 0.001). No statistically significant changes were found for the percentage of people who suffer from sleep bruxism, while the percentage of people who snored during lockdown decreased from 24.1% to 19.7% (*p* < 0.001).

Table 3 shows an analysis of the changes in lifestyle by gender during the COVID-19 lockdown. No association was reported by gender in perceiving changes in body weight, health concerns, diet quality changes, amount of physical activity changes, or sleep habit changes. In addition, 86.7% of the participants reported that they did not smoke before or during the lockdown (*p* < 0.001). 

Table 4 shows an analysis of changes in lifestyle by age group during the COVID-19 lockdown. No association was reported by age group, neither in the perception of diet quality changes nor smoking frequency changes. Additionally, 28.2% of participants older than 36 years old reported losing weight during the lockdown (*p* = 0.045), they were very worried about their health (*p* = 0.045), and a significant decrease in physical activity was found during lockdown (*p* = 0.028).

## 4. Discussion

One of the strategies of the Mexican government to avoid the massive contagion of COVID-19 was to establish “The Healthy Distance Campaign”, where non-essential face-to-face school and work activities were suspended, causing the population to stay at home and avoid going out into the streets, which could change their diet and lifestyle. This study was carried out to determine the effect of the COVID-19 lockdown on lifestyle changes in a sample of Mexicans, estimated by an online questionnaire. The main changes observed were increases in the frequency of the consumption of meat, vegetables, fruits, eggs and alcoholic drinks. Additionally, a significant decrease in the consumption of fast food, processed meat products, and alcoholic drinks was reported. This healthy eating habit was also reported in countries such as Kuwait and Italy, which increased the consumption of fruits and vegetables during lockdown.

In Spain, during the lockdown due to the pandemic, the trend was towards a greater consumption of fruits and vegetables and a decrease in the consumption of alcoholic drinks [9,14,15]. Furthermore, it has been reported that optimal nutrition may help to improve well-being and mitigate the risk and morbidity associated with the COVID-19 [13]. Similar recommendations were made in the 1918 influenza pandemic, suggesting that good nutrition, hygiene, and rest could help maintain health [16]. The Food and Agriculture Organization of the United Nations (FAO, Rome, Italy) was one of the first international organizations to publish a healthy diet guide during the COVID-19 pandemic. This guide recommends consuming fresh foods such as fruits, vegetables, whole grains, low-fat dairy, and healthy fats (olive oil and fish oil) and limiting the intake of sugar-sweetened beverages and processed foods high in calories and salt to improve the immune system [17]. The respondents of this study reported that a healthy food change during the COVID-19 lockdown could protect them against possible infection by COVID-19. Moreover, during that period, without effective medical treatments, the increased consumption of fruits and vegetables was the cheaper and more easily preventive method [9]. Another important fact about the diet during the COVID-19 lockdown in México is that although the trend was towards the consumption of healthy choices of food, it was observed that the number of soft drinks and desserts consumed did not change. The same behavior was observed in Poland, where the consumption of these products rich in sugars increased [18]; these foods contain carbohydrates that can reduce stress and produce serotonin that can positively affect mood. However, these foods have been associated with a higher probability of developing obesity and cardiovascular diseases, which have been shown to increase the risk of severe complications from COVID-19 [19,20]. On the other hand, Spain, during the lockdown, modified its eating behavior and other determinants of food consumption in a differentiated way based on individual and domestic factors [21]. Adherence to a Mediterranean diet has also been proposed, finding favorable results, determining that a healthy diet should be part of a comprehensive, multifactorial, individual risk strategy during a pandemic such as COVID-19 [22]. Regarding the physical activity reported by the participants of this study, a significant increase was observed in the percentage of people who stopped doing physical activity during the lockdown. The practice of yoga/Pilates was the only type of activity performed more frequently during this period; this phenomenon was also reported in the Italian lockdown during COVID-19 [23], which could be due to the ease of doing it at home due to the restriction of participating in outdoor activities. Orlandi et al. [24] conducted a cross-sectional web-based survey in April 2020 on a general population sample residing in Italy. Metabolic equivalents (hours/week) were used to evaluate physical activity. A total of 2218 participants (761 men and 1457 women) agreed to participate in the study and completed the questionnaire. The survey found that women, compared to men, had a lower level of physical activity before the COVID-19 lockdown. During the lockdown, women experienced a 1.9% reduction in intense physical activity and a 4.9% reduction in walking (hours/week). Furthermore, a worsening of sleep and stool passage was documented. On the other hand, men experienced an increase in alcohol consumption. 

Working remotely and receiving online classes caused people to spend more than six hours a day in front of the screen for work or recreation. This behavior was also observed during the lockdown in other countries; in China, people spent more than four hours a day in front of the screen [7]. This increased screen use led to a sedentary lifestyle associated with a higher risk of obesity, type 2 diabetes, cerebrovascular diseases, and depression in adults [25,26]. Epidemiological studies have indicated that spending more than two hours a day in front of the screen is associated with an increased risk of depression, mainly in women, and a 48% increased mortality risk. Furthermore, spending more than four hours a day in front of the screen increases the risk of cardiovascular disease events by approximately 125% [27].

The length and quality of sleep suffered significant changes due to the COVID-19 lockdown. Before the COVID-19 lockdown, more than half of the respondents slept less than 5 h a day, and during the lockdown, they could sleep more hours; this might be related to the fact that the respondents did not invest time in commuting to their workplaces and schools. Although the respondents slept more hours a day during the lockdown, the quality of sleep was negatively affected by insomnia, leg pain or cramps, and nightmares; this poor quality of sleep can affect physical and mental health, as well as reduce resistance to infections of the immune system [28]. Opposite results were reported in a study carried out in Greece, where it was identified that only 6.1% of the respondents presented a poor quality of sleep. In comparison, 17.2% answered that sleep quality improved, and 56.5% of the respondents did not present changes in sleep quality [29]. Another significant change reported in sleep quality was that the percentage of people who snored decreased during the lockdown. It has been reported that the quality of sleep can be affected by the lack of physical activity, so spending prolonged periods at home could cause an increase in a sedentary lifestyle due to the decrease in daily physical activity [30].

In lifestyle during the lockdown caused by the COVID-19 pandemic, no statistically significant effect was found concerning the gender or age groups of the respondents with regard to concerns about diet. However, people over 35 years old engaged in less physical activity. Furthermore, in a study of 693 Spaniards, the study group found the exact relationship. They performed less physical activity during lockdown [31]. 

A total of 84.9% of the surveyed people reported that they did not smoke before or during lockdown, highlighting that women are less likely to smoke compared to men. This is an important fact because there are studies in which smokers are more vulnerable to contracting respiratory diseases caused by viruses. In addition, there is evidence that current and past smoking produces a more severe clinical form of COVID-19, increasing the risk of death [32,33]. In relation to physical activity, participants perceived that they performed less physical activity (42.8%). It is notable that of this percentage, 52.6% corresponded to women and only 39.0% to men. 

During the COVID-19 lockdown, Mexicans made various changes in their food habits and lifestyle; however, it is difficult to determine if those changes were conscious. Perhaps the more conscious changes were the food habits due to the guides that the authorities published during the lockdown. Beneficial food habits help maintain good health, and sugary or alcoholic drinks are harmful to health. Additionally, being in self-quarantine, people had more free time, but they could not go to parks, gyms, schools, or workplaces. Lockdown could generate anxiety and all the negative changes found in this study. Perhaps these changes were unconscious. More investigations are required to know the post-pandemic context of the Mexican population. Recently, Barchielli et al. [11] investigated the main 21st-century concerns and the related habit and mental health changes in a sample of people from central and southern Italy. They reported that the young adults had higher scores in preoccupation, change in habits, and willingness to change habits in the future related to COVID-19. Older adults were the least worried, they expressed less fear, and were less willing to change their habits in the future, while elders were the group that least changed their current habits. Additionally, they related the results to the high level of COVID-19 knowledge and containment measures, which could decrease concern about the infection 

## 5. Limitations of the Study

The nature of the COVID-19 pandemic restricted people from gathering in public places, causing the study’s main limitation. Because of this restriction, the online survey was the only way to collect information. This means that a population sector without internet access could not be included. Additionally, the surveys were given anonymously, making it impossible to verify the responses given by the participants; in Section 3.2, the possible responses to the questionnaire were simplified to reduce the length of the questionnaire, since it has been proven that the longer a questionnaire is, the more negative an effect it has on the response rate. Additionally, neither the presence of obesity nor eating disorders were determined in the study, nor was the information on infection with COVID-19 reported. Another potential limitation of the study was that the respondents were mainly females, although this is usual in online questionnaires [34]. An unequal distribution of regions was reported in the sample analyzed, and there was an inaccuracy of self-reported/self-perceived data: no amount of food intake was recorded (only frequency). All of these factors should be considered when generalizing the results.

## 6. Conclusions

This survey shows that during the COVID-19 lockdown, the Mexican population made positive changes in their food habits as they increased the consumption of fruits, vegetables, legumes and eggs, and negative changes such as the increase in consumption of fast food and alcoholic drinks. Lifestyle also had positive repercussions, such as a decrease in smoking and increased hours of sleep. However, the lockdown caused negative changes in sleep quality, physical activity, and an increase in sedentary lifestyle. This study made it possible to observe that more intense strategies need to be implemented to promote healthy habits to improve the population’s health during a pandemic crisis.

## Figures and Tables

**Table 1:** Demographic characteristics of study participants (*n* = 1004).

Characteristics	*n*	%
Gender		
Male	306	30.5
Female	698	69.5
Age group		
18–25 years	510	50.8
26–35 years	213	21.2
36–45 years	190	18.9
46–55 years	58	5.8
56 years and over	33	3.3
Education level		
Middle school	5	0.5
High school	132	13.1
Bachelor’s degree	658	65.5
Master’s degree	131	13.0
Ph.D.	78	7.8
Cohabitation during COVID-19 lockdown		
Alone	54	5.4
With family	779	77.6
With a couple	74	7.4
With relatives	76	7.6
With friends	11	1.1
Other people	10	1.0
Minors at home		
Yes	568	56.6
No	436	43.4
Region		
Northwest	59	5.9
Northeast	27	2.7
West	37	3.7
East	44	4.4
North Central	83	8.3
South Central	127	12.6
Southwest	609	60.7
Southeast	18	1.8

**Table 2:** Sleep disorders before and during COVID-19 lockdown (%).

Sleep Disorders	Time	Yes	No	*p*-Value
Insomnia	B	27.4	72.6	<0.001
	D	56.3	43.7	
Nocturnal leg cramps	B	12.5	87.5	<0.001
	D	17.8	82.2	
Snoring	B	24.1	75.9	<0.001
	D	19.7	80.3	
Nightmare	B	10.4	89.6	<0.001
	D	18.5	81.5	
Sleep bruxism	B	9.4	90.6	0.450
	D	8.7	91.3	
None	B	75.4	24.6	<0.001
	D	87.1	12.9	

**Table 3:** Additional questions on health and lifestyle changes during COVID-19 lockdown by gender (*n* = 1004).

Variables	All *n* = 278	Sex Group			
		Male (*n* = 85)	Female *(n* = 193)	*p*-Value	*d*
	Weight perception during COVID-19 lockdown, *n* (%)				
Lost weight	66 (23.7)	21 (24.7)	45 (23.3)	0.457	
Increase	105 (37.8)	28 (32.9)	77 (39.9)	0.167	
Same as before	104 (37.4)	36 (42.4)	68 (35.2)	0.160	
Pregnant	3 (1.1)		3 (1.6)		
	How concerned were you about your health during COVID-19 lockdown, *n* (%)				
Very worried	55 (19.8)	18 (21.2)	37 (19.2)	0.407	0.012
Worried	129 (46.4)	37 (43.5)	92 (47.7)	0.306	
Little worried	74 (26.6)	24 (28.2)	50 (25.9)	0.395	
Nothing worried	20 (7.2)	6 (7.1)	14 (7.3)	0.587	
	How you consider your diet during COVID-19 lockdown, *n* (%)				
Healthier	132 (47.5)	39 (45.9)	93 (48.2)	0.412	0.096
Same as before	106 (38.1)	30 (35.3)	76 (39.4)	0.306	
Less healthy	40 (14.4)	16 (18.8)	24 (12.4)	0.114	
	How would you rate you smoking behavior during COVID-19 lockdown, *n* (%)				
Started smoking	3 (1.1)	2 (2.4)	1 (0.5)	0.22	0.316
Decreased	10 (3.6)	7 (8.2)	3 (1.6)	0.011	
Same as before	11 (4.0)	8 (9.4)	3 (1.6)	0.004	
Increased	6 (2.2)	2 (2.4)	4 (2.1)	0.594	
Stopped smoking	7 (2.5)	3 (3.5)	4 (2.1)	0.365	
I do not smoke	241 (86.7)	63 (74.1)	178 (92.2)	<0.001	
	How would you rate your physical activity during COVID-19 lockdown, *n* (%)				
Less physical activity	108 (38.8)	37 (43.5)	71 (36.8)	0.176	0.268
Same as before	126 (45.3)	39 (45.9)	87 (45.1)	0.502	
More physical activity	44 (15.8)	9 (10.6)	35 (18.1)	0.076	
	How would you rate your sleep routine, *n* (%)				
I sleep the same	229 (82.4)	67 (78.8)	162 (83.9)	0.194	0.315
I sleep earlier	21 (7.6)	9 (10.6)	12 (6.2)	0.153	
I sleep later	121 (43.5)	39 (45.9)	82 (42.5)	0.346	
My sleep is interrupted more often	32 (11.5)	6 (7.1)	26 (13.5)	0.087	
I take more naps than usual	16 (5.8)	3 (3.5)	13 (6.7)	0.223	
I have a very changeable sleep schedule	39 (14.0)	10 (11.8)	29 (15.0)	0.301	

*p*-value for overall difference was calculated from Chi–Square tests at the 5% level; *d*, Cohen’s effect size.

**Table 4:** Additional questions on health and lifestyle changes during COVID-19 lockdown by age (*n* = 1004).

Variables	All *n* = 278	Age Group (Years)			
		18–35 (*n* = 200)	≥36 *(n* = 78)	*p*-Value	*d*
	Weight perception during COVID-19 lockdown, *n* (%)				
Lost weight	58 (20.9)	36 (18.0)	22 (28.2)	0.045	0.022
Increase	115 (41.4)	78 (39.0)	37 (47.4)	0.126	
Same as before	104 (37.4)	85 (42.5)	19 (24.4)	0.003	
Pregnant	1 (0.4)	1 (0.5)	0 (0.0)	0.719	
	How concerned were you about your health during COVID-19 lockdown, *n* (%)				
Very worried	58 (20.9)	36 (18.0)	22 (28.2)	0.045	0.100
Worried	128 (46.0)	101 (50.5)	27 (34.6)	0.012	
Little worried	64 (23.0)	47 (23.5)	17 (21.8)	0.447	
Nothing worried	28 (10.1)	16 (8.0)	12 (15.4)	0.056	
	How you consider your diet during COVID-19 lockdown, *n* (%)				
Healthier	117 (42.1)	79 (39.5)	38 (48.7)	0.103	0.199
Same as before	108 (38.8)	83 (41.5)	25 (32.1)	0.094	
Less healthy	53 (19.1)	38 (19.0)	15 (19.2)	0.544	
	How would you rate you smoking behavior during COVID-19 lockdown, *n* (%)				
Started smoking	3 (1.1)	1 (0.5)	2 (2.6)	0.191	0.079
Decreased	11 (4.0)	10 (5.0)	1 (1.3)	0.136	
Same as before	12 (4.3)	7 (3.5)	5 (6.4)	0.223	
Increased	6 (2.2)	6 (3.0)	0 (0)	0.136	
Stopped smoking	8 (2.9)	7 (3.5)	1 (1.3)	0.292	
I do not smoke	238 (85.6)	169 (84.5)	69 (88.5)	0.260	
	How would you rate your physical activity during COVID-19 lockdown, *n* (%)				
Less physical activity	119 (42.8)	78 (39.0)	41 (52.6)	0.028	0.078
Same as before	125 (45.0)	100 (50.0)	25 (32.1)	0.005	
More physical activity	34 (12.2)	22 (11.0)	12 (15.4)	0.210	
	How would you rate your sleep routine, *n* (%)				
I sleep the same	55 (19.8)	35 (17.5)	20 (25.6)	0.088	0.030
I sleep earlier	19 (6.8)	13 (6.5)	6 (7.7)	0.451	
I sleep later	119 (42.8)	91 (45.5)	28 (35.9)	0.093	
My sleep is interrupted more often	31 (11.2)	21 (10.5)	10 (12.8)	0.359	
I take more naps than usual	20 (7.2)	14 (7.0)	6 (7.7)	0.510	
I have a very changeable sleep schedule	34 (12.2)	26 (13.0)	8 (10.3)	0.343	

*p*-value for overall difference was calculated from Chi–Square tests at 5% level; *d*, Cohen’s effect size.

## Data Availability

Not applicable.

## Supplementary Materials

The following supporting information can be downloaded at: https://www.mdpi.com/article/10.3390/healthcare10122537/s1, Table S1. The frequency of the consumption of select products before (B) and during (D) COVID-19 lockdown (%).
