# Supplementary material for: Lifestyle Changes among Mexican People during the COVID-19 Lockdown in 2020: A Cross-Sectional Study
Source: Healthcare (Basel). 2022 Dec 14;10(12):2537. doi: 10.3390/healthcare10122537 (PMC9778622; doi:10.3390/healthcare10122537)
Supplement: Supplementary file 1 [file healthcare-10-02537-s001.zip › healthcare-1961973-supplementary.pdf]

healthcare

MDPI

Table S1. The frequency of the consumption of select products before (B) and during (D) COVID-19 lockdown (%).

**Food items**	**Time**	*< 1 day per week*	*1-2 days per week*	*3-4 days per week*	*5-6 days per week*	*7 days per week*
Meat	B	1.7	38.9	39.0	14.8	5.5
	D	1.9	35.4	39.3	17.3	6.1
Vegetable	B	1.2	23.2	36.4	21.4	17.8
	D	0.5	16.6	35.5	25.2	22.2
Fruit	B	1.7	26.2	31.3	20.6	20.2
	D	0.8	19.4	30.8	23.7	25.3
Eggs	B	2.0	34.3	39.8	17.2	6.7
	D	1.7	31.2	38.6	19.2	9.3
Cereals	B	6.2	35.5	27.6	15.7	15.0
	D	6.4	31.9	30.7	16.2	14.8
Legumes	B	4.1	33.8	41.0	14.5	6.6
	D	4.2	32.1	38.6	17.1	8.0
Dairy products	B	3.7	26.5	28.9	22.4	18.5
	D	4.6	28.8	27.8	19.9	18.9
Water	B	1.4	4.5	6.1	22.4	65.6
	D	1.8	4.8	5.7	19.6	68.1
Sweetened beverages	B	20.0	44.9	20.4	8.5	6.2
	D	23.0	45.5	18.5	7.8	5.2
Sweeteners	B	13.5	52.5	23.7	7.1	3.2
	D	15.0	52.9	21.4	7.6	3.1
Fast food	B	15.9	54.7	18.1	7.9	3.4
	D	22.8	52.7	16.1	6.2	2.2
Alcoholic drinks	B	43.2	45.0	6.4	3.6	1.8
	D	49.2	38.4	6.2	3.9	2.3
Processed meat products	B	15.7	47.8	23.9	10.3	2.3
	D	17.4	51.6	18.7	9.7	2.6
Butter	B	19.0	52.8	18.2	7.0	3.0
	D	17.1	51.6	19.1	9.0	3.2
Oils and margarines	B	4.1	33.2	32.1	19.2	11.5
	D	4.6	37.5	26.1	21.0	10.8
